# The transcription factor SbbHLH168 enhances salt tolerance by coordinating ion homeostasis and lignin content in sorghum

**DOI:** 10.1007/s44154-026-00330-4

**Published:** 2026-07-21

**Authors:** Simin Li, Zishuo Han, Rui Liu, Zengting Chen, Xuemei Wang, Na Sui, Zhiying Zhao

**Affiliations:** 1 Shandong Provincial Key Laboratory of Plant Stress Biology and Genetic Improvement, College of Life Sciences, Shandong Normal University, Jinan, 250014 China; 2 National Center of Technology Innovation for Comprehensive Utilization of Saline-Alkali Land, Dongying, 257347 China

**Keywords:** Salt stress, SbbHLH168, SbbHLH35, Lignin content, Sorghum

## Abstract

Soil salinization is an escalating global threat to crop productivity. Here, we demonstrate that the sorghum bHLH transcription factor SbbHLH168 is a central regulator of salt-stress tolerance. SbbHLH168 is nuclear-localized, constitutively expressed in all organs and rapidly up-regulated by NaCl. Overexpression of *SbbHLH168* in either Arabidopsis or sorghum conferred markedly higher biomass retention, longer roots, lower relative electrical conductivity, less accumulation of superoxide and H₂O₂, and lower Na⁺/K⁺ ratio through decreased Na⁺ accumulation under salt stress, whereas VIGS silent groups displayed the opposite phenotype. qRT-PCR and biochemical assays demonstrated that SbbHLH168 up-regulates key lignin-biosynthetic genes (*SbC4H*, *SbF5H*, *SbCOMT1*) and increases root lignin content under salt stress, thereby reinforcing cell-wall integrity and limiting ion leakage. Furthermore, yeast two-hybrid and BiFC assays confirmed that SbbHLH168 interacts with SbbHLH35 in the nucleus, indicating that SbbHLH168's functional activity depends on heterodimerization with SbbHLH35. Collectively, SbbHLH168 integrates ion homeostasis, ROS scavenging and lignin content to confer salt tolerance, providing a direct gene target for molecular breeding of resilient sorghum cultivars.

**Supplementary Information:**

The online version contains supplementary material available at 10.1007/s44154-026-00330-4.

## Introduction

In recent years, global saline-alkali land area has exhibited a continuous expansion trend due to persistent temperature rise, freshwater scarcity, and excessive fertilizer application (Duchenne-Moutien et al. 2021). Soil salinization impedes crop nutrient uptake, disrupts normal growth and development, severely threatens global agricultural productivity, and compromises food security (Liang et al. 2024; Zhou et al. 2024).

Plants enhance salt tolerance through integrated mechanisms involving osmotic adjustment, ionic homeostasis, and reactive oxygen species (ROS) scavenging. First, under salt stress, plants activate the biosynthesis and accumulation of compatible osmolytes to maintain cell turgor, reduce cellular osmotic potential, thereby counteracting osmotic stress (Park et al. 2016). Second, plants regulate Na⁺ levels to mitigate salt stress damage via three key strategies: (i) vacuolar compartmentalization of Na⁺, (ii) cellular Na⁺ exclusion, and (iii) restriction of Na⁺ influx. For instance, the vacuolar membrane-localized Na⁺/H⁺ antiporter NHX1 transports Na⁺ from the cytoplasm into the vacuole while exporting H⁺ to the cytoplasm, maintaining ion equilibrium and preventing Na⁺ toxicity to cytoplasmic enzymes. Concurrently, the plasma membrane-localized Na⁺/H⁺ antiporter SOS1 (Salt Overly Sensitive 1, also designated NHX7) utilizes the H⁺-ATPase-generated proton gradient to extrude Na⁺ against its concentration gradient while importing H⁺ (Bassil et al. 2014; Yamaguchi et al. 2013). Furthermore, salt stress triggers ROS overaccumulation; plants counteract oxidative damage via enzymatic antioxidants (e.g., catalase decomposing H₂O₂ to H₂O, ascorbate peroxidase eliminating H₂O₂ through the ascorbate–glutathione cycle, superoxide dismutase regulating O₂⁻ and H₂O₂ levels) and non-enzymatic antioxidants (Schwacke et al. 2003).

Transcription factors (TFs) serve as master regulators in gene expression networks by binding *cis*-elements in promoter regions of target genes, thereby activating or repressing transcription to modulate plant growth and abiotic stress responses (Rabeh et al. 2025; Shu et al. 2024). Notably, several TFs critically regulate salt adaptation: OsbZIP71 binds to the promoter of *OsNHX1*, facilitating vacuolar sequestration of excess Na⁺/K⁺ to reduce cytoplasmic Na⁺ concentration (Liu et al. 2014); OsHBP1b enhances vacuolar membrane stability, increases K⁺/Na⁺ ratio, and elevates SOD activity to diminish H₂O₂ accumulation (Lakra et al. 2015); salt stress promotes phosphorylation of SOS2 kinase and stabilizes PLT1/2, core transcription factors regulating root apical meristem, thereby sustaining growth to aid resistance against salt stress (Hao et al. 2023).

The basic helix-loop-helix (bHLH) transcription factors constitute the second largest family of transcription factors in eukaryotes after MYB. They play a crucial role in plant responses to abiotic stresses, such as drought-induced water deficit, salt-mediated ion imbalance, and cold stress (Feller et al. 2011), offering novel approaches and targets for enhancing plant stress tolerance. For instance, *TabHLH39* overexpression in wheat elevates proline and soluble sugars, enhancing Arabidopsis tolerance to cold, drought, and saline stress (Zhai et al. 2016); *SlbHLH22* overexpression in tomato augments ROS scavenging and osmotic adjustment (Waseem et al. 2019); while wild rice OrbHLH001 binds the E-box in the *OsAKT1* promoter to regulate Na⁺/K⁺ homeostasis (Chen et al. 2013). Consequently, the bHLH family is pivotal for salt-alkali stress adaptation, yet its regulatory targets and molecular mechanisms demand deeper exploration.

Sorghum (*Sorghum bicolor* L.), a C4 cereal crop, has emerged as a strategic resource for sustainable agriculture and bioeconomy due to its high photosynthetic efficiency and environmental resilience (Dahlberg et al. 2019; Hao et al. 2021). Its stems serve as a prime feedstock for ethanol production, while grains provide starch and dietary fiber (Xie et al. 2019). Crucially, sorghum can preserve stomatal conductance, chlorophyll content, and photosynthetic capacity under salt stress (Johnson et al. 2021). In addition, sorghum possesses a compact genome, facilitating efficient identification of salt-tolerant genes and elucidation of conserved mechanisms. Genome-wide association studies (GWAS) confirm that sorghum germplasm harbors abundant salt-tolerant allelic variation, exemplified by the discovery of the major alkaline tolerance gene AT1 (Zhang et al. 2023). In summary, sorghum serves as a strategic model crop for salt stress research, though key regulatory genes and networks remain uncharacterized.

The current understanding of sorghum salt tolerance mechanisms involves the regulatory roles of multiple transcription factor families, primarily including WRKY, MYB, and bHLH that control ion transport, ABA signaling pathway, and stress-gene expression (Lee et al. 2022). For example, SbWRKY55 interacts with the zinc finger protein SbFYVE1 and binds to the promoter of *SbBGLU22* to inhibit its expression, negatively regulating ABA signaling and salt tolerance (Song et al. 2022a). SbMYBHv33 modulates ROS and ion dynamics to suppress salt tolerance (Zheng et al. 2023). SbbHLH85 promotes root hair development via ABA/auxin pathways, increasing Na⁺ uptake while decreasing Pi content (Song et al. 2022b). However, systematic studies on *SbbHLH* genes in sorghum salt adaptation are scarce, and their regulatory hierarchies require elucidation.

This study functionally characterizes the bHLH TF gene *SbbHLH168* in sorghum. Using heterologous expression in Arabidopsis, virus-induced gene silencing (VIGS), and *Agrobacterium rhizogenes*-mediated transformation, we generated *SbbHLH168* knockdown and overexpression plants. Physiological assays, protein interaction validation, and target gene identification collectively reveal *SbbHLH168*'s role in salt tolerance, providing theoretical foundations and gene targets for breeding salt-resilient sorghum.

## Results

### SbbHLH168 is a salt-induced bHLH transcription factor

**Fig. 1:**
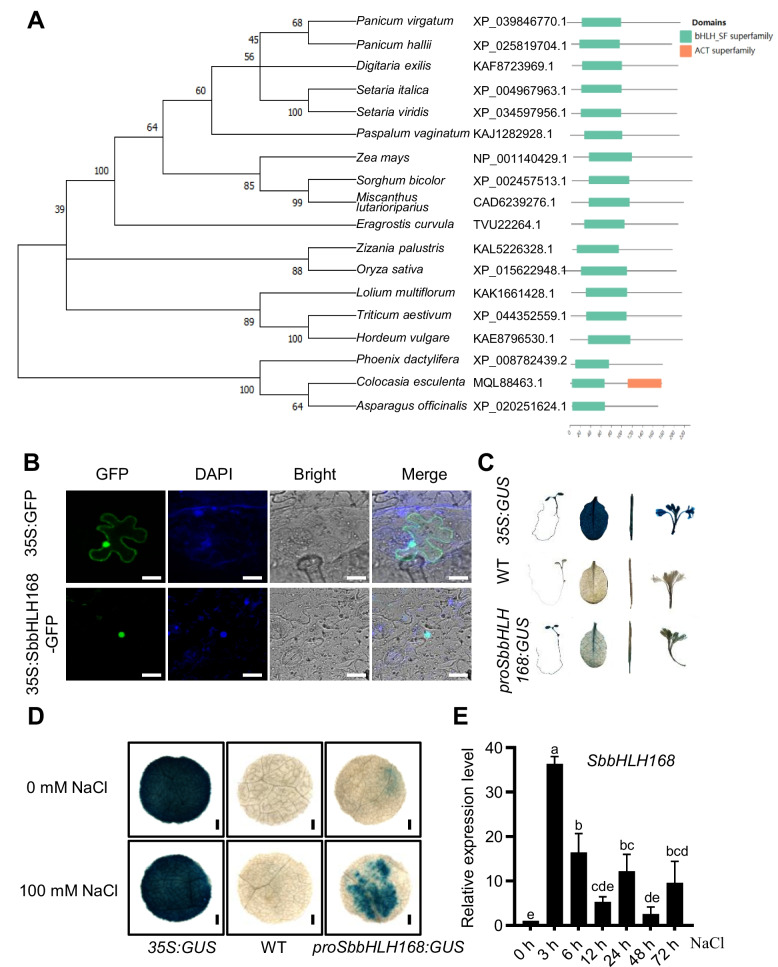
Functional characterization of the bHLH transcription factor SbbHLH168 in salt stress. **A** Phylogenetic analysis and domain architecture of bHLH168 homologs. Conserved domains were predicted by bHLH-SF superfamily (green boxes) and ACT superfamily (orange boxes). **B** Subcellular localization of SbbHLH168. Transient expression of 35S:SbbHLH68:GFP and 35S:GFP was performed in tobacco leaves. Localization of cell nucleus labeled with DAPI dye. Scale bar = 25 μm. **C** Tissue-specific expression of SbbHLH68. Transgenic Arabidopsis plants carrying the *proSbbHLH168:GUS* construct were stained for GUS activity. Wild-type (WT, Col-0) served as the negative control, while *35S:GUS* Arabidopsis served as the positive control. **D** Salt-induced expression of *SbbHLH168* by GUS staining. *proSbbHLH168:GUS* construct-transformed tobacco leaves, observed for GUS activity after 12 h of treatment with 100 mM NaCl. Scale bar = 1 cm. **E** Expression of *SbbHLH168* under different NaCl treatment times in sorghum roots. Error bars indicate mean ± standard deviation (SD), *n* = 3. Statistically significant differences are indicated by different letters (*p* < 0.05)

The published results from the laboratory revealed that *SbbHLH168* is highly expressed in the roots of salt-tolerant sorghum varieties (Yang et al. 2018). SbbHLH168 encodes 232 amino acids, with the HLH domain (residues 37–106) being highly conserved and forming a typical bHLH fold (Fig. S1). Its three-dimensional structure adopts a V-shaped dimer, where two pairs of α-helices interlock through hydrophobic surfaces (Fig. S1C), suggesting it relies on homo- or heterodimerization to achieve transcriptional regulatory functions. Phylogenetic analysis of bHLH168 homologs across 15 Poaceae species and 3 outgroup species revealed that SbbHLH168 forms a monophyletic clade with its orthologs from *Zea mays* and *Miscanthus lutarioriparius*, indicating closest evolutionary relationships (Fig. 1A and S1D). All identified bHLH168 homologs contained the conserved N-terminal bHLH-SF domain characteristic of this transcription factor family. Subcellular localization confirmed that the SbbHLH168-GFP fusion protein exclusively accumulated in the nucleus, as demonstrated by co-localization with DAPI staining (Fig. 1B). Tissue-specific expression analysis using *proSbbHLH168:GUS* transgenic Arabidopsis revealed ubiquitous GUS activity in roots, leaves, siliques, and inflorescences (Fig. 1C). Consistent with this, analysis of publicly available sorghum transcriptome data detected *SbbHLH168* transcripts across all examined tissues and developmental stages (Chen et al. 2025; Fig. S2A), and qRT-PCR validation confirmed its broad expression in sorghum, with the highest levels in leaf and root at the three-leaf stage (Fig. S2B). Thus, SbbHLH168 is a nucleus-localized transcription factor broadly expressed throughout sorghum development.

To determine whether *SbbHLH168* expression is salt-responsive, we transiently expressed *proSbbHLH168:GUS* in *N. benthamiana* leaves. Salt treatment significantly increased *proSbbHLH168:GUS* activity compared to untreated control (Fig. 1D). Consistent with this, qRT-PCR analysis demonstrated rapid induction of *SbbHLH168* transcripts in sorghum under salt stress, with expression peaking at 3 h (Fig. 1E). Collectively, these data indicate that SbbHLH168 functions as a salt-inducible transcription factor.

### Overexpression of *SbbHLH168* enhances salt tolerance in Arabidopsis

**Fig. 2:**
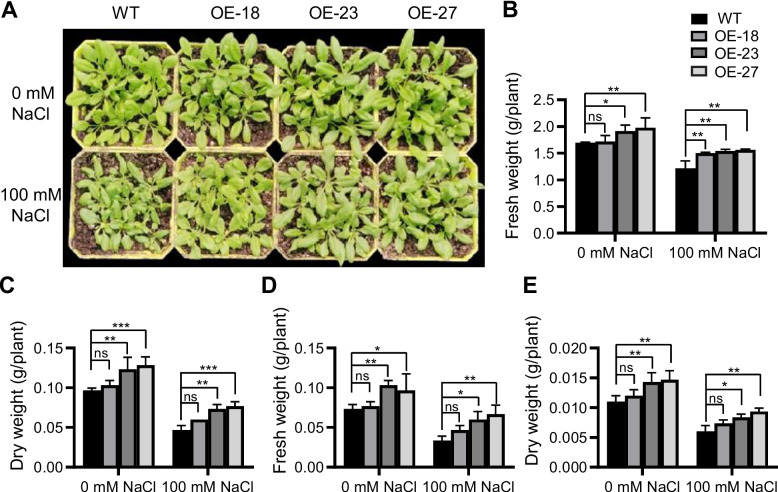
Overexpression of SbbHLH168 enhances salt tolerance in Arabidopsis under salt stress. **A** Phenotypes of WT and SbbHLH168-OE Arabidopsis seedlings (OE-18, OE-23, OE-27) under 0 mM and 100 mM NaCl. **B–E** Measurements of shoot fresh weight (**B**), shoot dry weight (**C**), root fresh weight (**D**), and root dry weight (**E**) of WT and SbbHLH168-OE lines with and without salt treatment shown in (**A**). Error bars indicate mean ± standard deviation (SD) of *n* = 3. Statistically significant differences are indicated by asterisks (two-way ANOVA, ns *p* > 0.05, * *p* < 0.05, ** *p* < 0.01, *** *p* < 0.0005)

To evaluate the salt tolerance conferred by SbbHLH168, SbbHLH168-overexpressing (OE) Arabidopsis lines (OE-18, OE-23, OE-27; Fig. S3A) were subjected to salt stress. Under 100 mM NaCl treatment, SbbHLH168-OE exhibited greener leaves and more robust growth compared to wild-type (WT) (Fig. 2A). Biomass quantification confirmed superior performance in SbbHLH168-OE: shoot fresh weight, shoot dry weight, root fresh weight, and root dry weight were significantly higher in OE lines than in WT under salt stress (Fig. 2B–E), indicating enhanced biomass retention. Root length measurements further revealed reduced salt sensitivity in SbbHLH168-OE relative to WT (Fig. S3B, C).

**Fig. 3:**
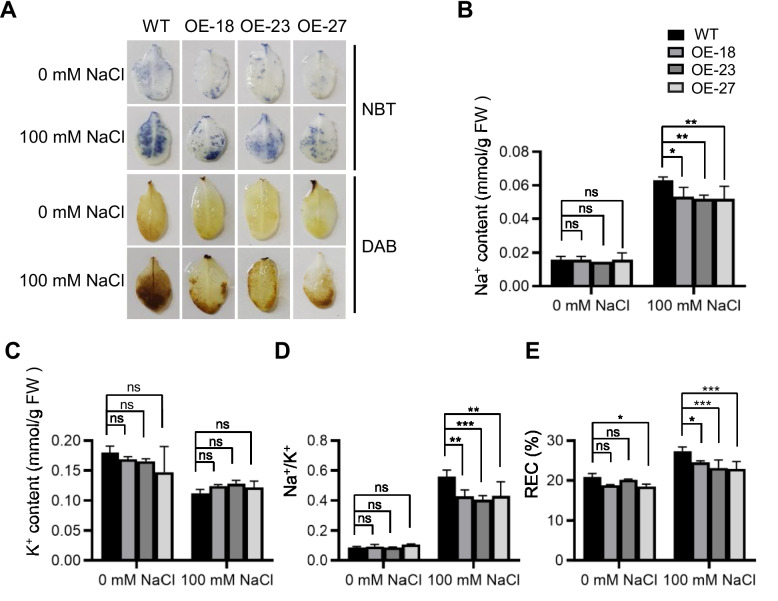
Overexpression of *SbbHLH168* reduces ROS accumulation and regulates ion levels in transgenic Arabidopsis. **A** NBT and DAB staining of SbbHLH168-OE transgenic Arabidopsis leaves under 0 mM and 100 mM NaCl. **B–E** Measurements of Na^+^ content (**B**), K^+^ content (**C**), Na^+^/K.^+^ ratio (**D**), and REC (**E**) of WT and SbbHLH168-OE transgenic Arabidopsis with and without salt treatment. Error bars indicate mean ± standard deviation (SD) of *n* = 3. Statistically significant differences are indicated by asterisks (two-way ANOVA, ns *p* > 0.05, * *p* < 0.05, ** *p* < 0.01, *** *p* < 0.0005)

NBT (Nitro blue tetrazolium) and DAB (3,3'-Diaminobenzidine) staining revealed that while all genotypes showed increased ROS accumulation under salt stress, SbbHLH168-OE displayed less intense staining than WT (Fig. 3A, B), consistent with enhanced ROS scavenging capacity. Ion homeostasis analysis showed that SbbHLH168-OE accumulated significantly less Na⁺ content (Fig. 3C) and maintained a lower Na⁺/K⁺ ratio (Fig. 3D) than WT under stress, with no significant difference in K⁺ content (Fig. 3C). Additionally, relative electrical conductivity was reduced in SbbHLH168-OE (Fig. 3E). Collectively, these results demonstrate that SbbHLH168 overexpression enhances salt tolerance in Arabidopsis by improving antioxidant capacity, ion homeostasis, and membrane stability.

### SbbHLH168 enhances salt tolerance in sorghum

**Fig. 4:**
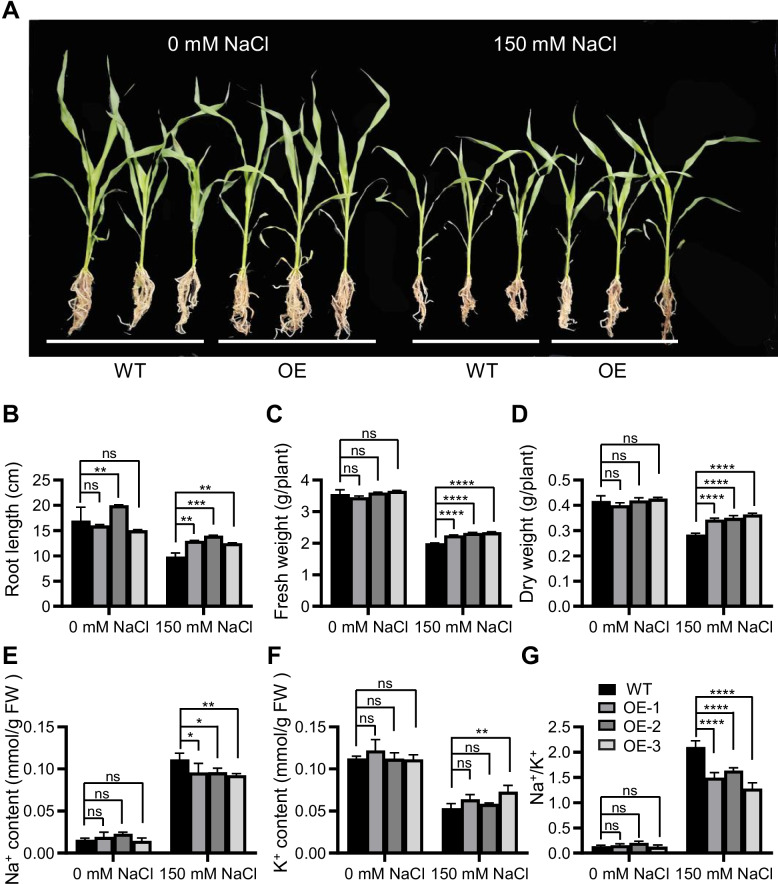
Overexpression of *SbbHLH168* enhances salt tolerance in sorghum. **A** Phenotypes of WT and SbbHLH168-OE transgenic sorghum under 0 mM and 150 mM NaCl. **B–G** Measurements of root length (**B**), root fresh weight (**C**), and root dry weight (**D**), Na^+^ content (**E**), K^+^ content (**F**), and Na^+^/K.^+^ ratio (**G**) of WT and SbbHLH168-OE with and without salt treatment. Error bars indicate mean ± standard deviation (SD) of *n* = 3. Statistically significant differences are indicated by asterisks (two-way ANOVA, ns *p* > 0.05, * *p* < 0.05, ** *p* < 0.01, *** *p* < 0.0005, **** *p* < 0.0001)

To further assess the salt tolerance of *SbbHLH168* in sorghum, we generated SbbHLH168-OE sorghum via *A. rhizogenes*-mediated hairy root transformation (Fig. S4). Under 150 mM NaCl treatment for 7 days, three independent SbbHLH168-OE groups with the highest expression (OE-1, OE-2, OE-3) exhibited more robust growth compared to controls (Fig. 4A). This was evidenced by significantly longer roots (Fig. 4B), increased fresh weight (Fig. 4C), and dry weight (Fig. 4D). At the ionic level, SbbHLH168-OE exhibited lower Na⁺ content and Na⁺/K⁺ ratio than WT after salt treatment, while K⁺ content was slightly higher than WT (Fig. 4E-G).

**Fig. 5:**
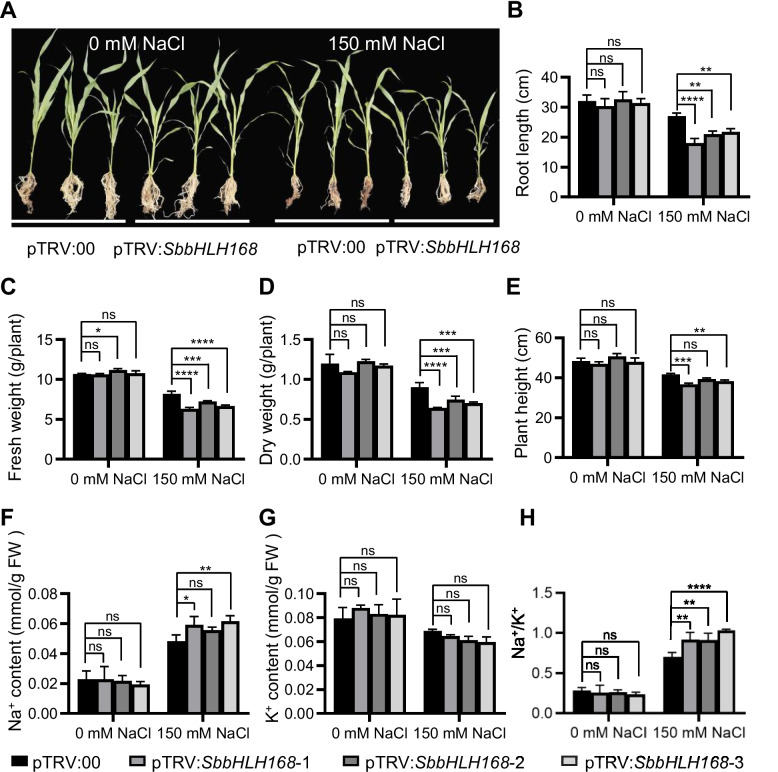
pTRV:*SbbHLH168* silenced plants reduce salt tolerance in sorghum. **A** Phenotypes of pTRV:00 and pTRV:*SbbHLH168* silenced plants under 0 mM and 150 mM NaCl in sorghum. **B–H** Measurements of root length (**B**), root fresh weight (**C**), root dry weight (**D**), and plant height (**E**), Na^+^ content (**F**), K^+^ content (**G**), and Na^+^/K.^+^ ratio (**H**) of pTRV:00 and pTRV:*SbbHLH168* silenced plants with and without salt treatment. Error bars indicate mean ± standard deviation (SD) of *n* = 3. Statistically significant differences are indicated by asterisks (two-way ANOVA, ns *p* > 0.05, * *p* < 0.05, ** *p* < 0.01, *** *p* < 0.0005, **** *p* < 0.0001)

Conversely, VIGS-mediated silencing of *SbbHLH168* in sorghum (pTRV:*SbbHLH168*; Fig. S5) resulted in enhanced salt sensitivity (Fig. 5A). Under salt stress, all three silenced groups showed significant reductions in root length (Fig. 5B), fresh weight (Fig. 5C), and dry weight (Fig. 5D) compared with pTRV:00. For plant height (Fig. 5E) and Na⁺ content (Fig. 5F), pTRV:*SbbHLH168-*1 and −3 exhibited significant changes, whereas pTRV:*SbbHLH168-*2 did not differ significantly from the control. K⁺ content (Fig. 5G) showed a mild downward trend across all three silenced groups but did not reach significance in any group. Notably, the Na⁺/K⁺ ratio (Fig. 5H) was significantly elevated in all three silenced groups relative to pTRV:00, indicating that SbbHLH168 enhances salt tolerance in sorghum by modulating ion homeostasis.

### SbbHLH168 interacts with SbbHLH35

**Fig. 6:**
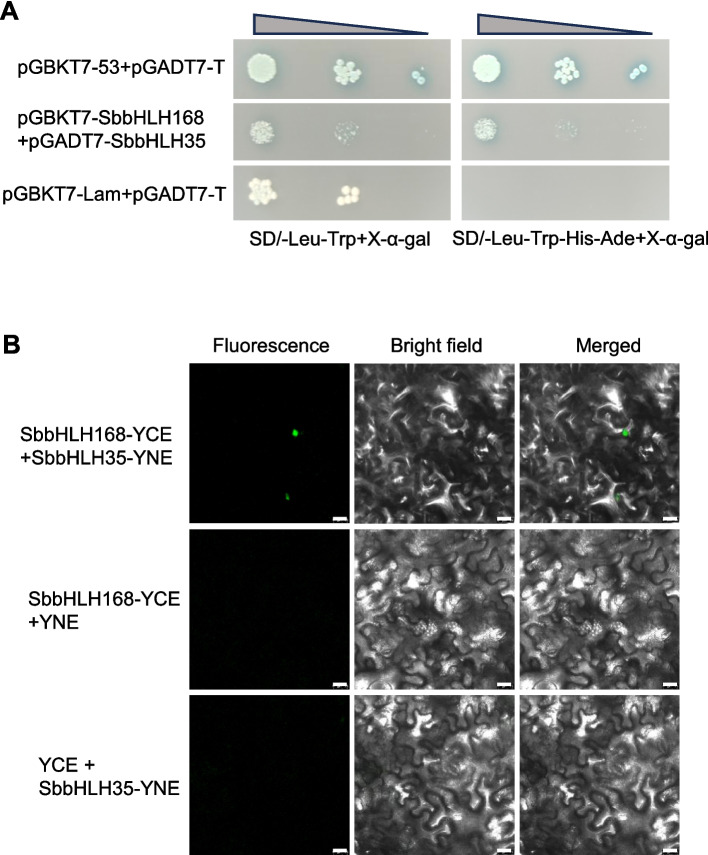
SbbHLH168 interacted with SbbHLH35. **A** Y2H assays of the interaction between SbbHLH168 and SbbHLH35. The positive interactions were evaluated by yeast cells grown on SD/-Leu/-Trp/-His/-Ade/X-α-gal medium. pGBKT7-53 + pGADT7-T served as the positive control group, while pGBKT7-Lam + pGADT7-T served as the negative control group. **B** BiFC assays of the interaction between SbbHLH168 and SbbHLH35. Transient expression of SbbHLH168-YCE (C-terminal part of YFP) and SbbHLH35-YNE (N-terminal part of YFP) was achieved in tobacco leaves. Scale bar = 25 μm

Structural analysis of SbbHLH168 suggested potential transcriptional regulation via homodimerization or heterodimerization (Fig. S1). To identify interacting partners, we performed yeast two-hybrid screening. Initial autoactivation tests confirmed that pGBKT7-SbbHLH168 exhibited no self-activating activity, but positive controls (pGBKT7-53 + pGADT7-T) grew normally under selective conditions (Fig. S6). When pGBKT7-SbbHLH168 (bait) and pGADT7-SbbHLH35 (prey) were co-transformed into quadruple dropout medium (-Leu/-Trp/-His/-Ade), they grew robust and produced blue coloration with X-α-Gal (Fig. 6A), indicating interaction between SbbHLH168 and SbbHLH35. To investigate whether *SbbHLH35* expression responds to salt stress, we performed qRT-PCR analysis. *SbbHLH35* was significantly upregulated in WT after NaCl treatment (Fig. S7A). Notably, the salt-tolerant cultivar M-81E exhibited markedly higher basal expression of *SbbHLH35* compared with the salt-sensitive cultivar Roma (Yang et al. 2018; Fig. S7B), suggesting a potential role of SbbHLH35 in salt tolerance. Furthermore, BiFC revealed that strong YFP fluorescence confined exclusively to the nucleus was observed only when SbbHLH168-YCE and SbbHLH35-YNE were co-expressed, whereas the negative control exhibited no fluorescence (Fig. 6B). These results indicate that SbbHLH168 and SbbHLH35 exhibit specific interactions both in vitro and in vivo.

### SbbHLH168 regulates lignin biosynthesis to enhance salt tolerance

**Fig. 7:**
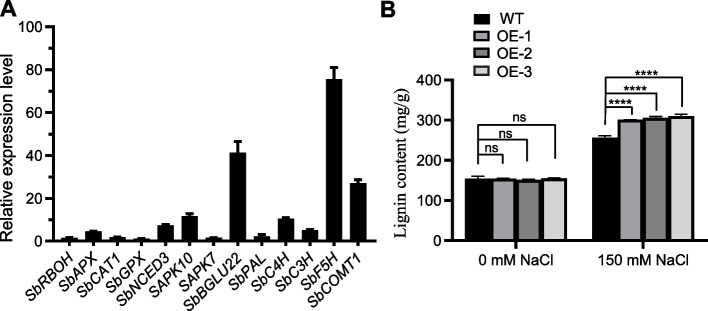
SbbHLH168 promotes lignin accumulation under salt stress. **A** Expression of ABA-related genes and lignin synthesis genes in SbbHLH168-OE transgenic sorghum compared to WT under salt stress. **B** Lignin content of WT and SbbHLH168-OE plants of sorghum under salt stress. Error bars indicate mean ± standard deviation (SD) of *n* = 3. Statistically significant differences are indicated by asterisks (two-way ANOVA, ns *p* > 0.05, **** *p* < 0.0001)

To identify potential downstream targets of SbbHLH168, we analyzed the expression of stress-related genes in salt-treated SbbHLH168-OE sorghum and WT via qRT-PCR. Among ABA signaling pathway-related genes, *SbNCED3*, *SAPK10*, and *SbBGLU22* were significantly upregulated in OE groups compared to WT under salt stress, with *SbBGLU22* showing the highest fold increase (Fig. 7A).

Additionally, key lignin biosynthesis genes (*SbC4H*, *SbF5H*, and *SbCOMT1*) exhibited significantly elevated expression in SbbHLH168-OE, with *SbF5H* demonstrating the most pronounced upregulation (Fig. 7A). Lignin content measurements revealed no significant difference between SbbHLH168-OE and WT under control conditions. However, after 150 mM NaCl treatment, lignin content increased in all groups, with SbbHLH168-OE exhibiting a greater magnitude of increase than WT (Fig. 7B). These results suggest that SbbHLH168 enhances salt tolerance by regulating lignin biosynthetic genes, thereby increasing lignin accumulation under stress conditions.

## Discussion

Soil salinization is a global threat to crop production, and salt stress reduces yields across major cereals by impairing growth and development (Sui et al. 2017; Asano et al. 2012). Sorghum tolerates saline conditions better than most staple crops, making it a logical candidate for cultivation on marginal lands affected by soil salinity. However, the molecular mechanisms underpinning sorghum's salt tolerance remain incompletely understood. Here, we functionally characterize the bHLH transcription factor SbbHLH168, and we show that it enhances salt tolerance by simultaneously regulating ion homeostasis, ROS scavenging, and lignin biosynthesis.

### SbbHLH168 acts as a key positive regulator under salt stress

The bHLH family, the second largest transcription factor family in plants, controls diverse abiotic stress responses (Jiang et al. 2009). Characterized members improve stress tolerance through ion transport (e.g., OsbHLH001), ROS scavenging and osmotic adjustment (e.g., SlbHLH22), and proline accumulation (e.g., TabHLH39) (Chen et al. 2013; Waseem et al. 2019; Zhai et al. 2016). Despite their functional breadth, systematic study of the bHLH family in sorghum lags behind that in rice and Arabidopsis. Only a handful of sorghum bHLH members have been examined under salt stress, most notably SbbHLH85, which plays a negative regulatory role—it promotes root hair elongation, increases Na⁺ uptake via ABA and auxin signaling, and exacerbates ionic stress (Song et al. 2022b). SbbHLH168, by contrast, acts as a positive regulator: overexpression confers enhanced salt tolerance, whereas silencing produces salt-hypersensitive phenotypes. These two sorghum paralogs thus occupy opposite ends of the functional spectrum, underscoring that shared domain architecture alone does not predict physiological output even within a single species.

Pairwise alignment of SbbHLH168 against 17 homologs (15 Poaceae and 3 non-Poaceae outgroups) (Fig. 1A and S1D) reveals a phylogenetic gradient in sequence similarity: highest within Andropogoneae (MlbHLH168 from *Miscanthus lutarioriparius*, score 88.60), moderate across the broader Panicoideae (72.15–78.08), and sharply reduced in Pooideae (52.59–57.33), Oryzoideae (OsbHLH168, 50.00), and non-Poaceae outgroups (50–52). The bHLH-SF domain is the most conserved region, particularly the basic region and Helix 1, whereas flanking regions that mediate protein–protein interactions are considerably more variable (Fig. S1D). Consequently, DNA-binding specificity may be broadly conserved across orthologs, but interaction surfaces—and therefore downstream regulatory networks—are likely to have diverged. The notably low similarity between SbbHLH168 and OsbHLH168 (50.00, indistinguishable from non-Poaceae outgroups) provides a sequence-level basis for the functional contrast between SbbHLH168 and the rice ortholog OsbHLH034 discussed below.

SbbHLH168 localizes to the nucleus (Fig. 1D), groups phylogenetically with Andropogoneae orthologs (Fig. 1A), and displays constitutive expression across all organs examined (Fig. 1C, S2). Its transcript level was rapidly and significantly induced by NaCl (Fig. 1E). Overexpression of SbbHLH168 enhanced salt tolerance in both Arabidopsis and sorghum (Figs. 2A-E, 4A-D), whereas VIGS-mediated silencing compromised tolerance (Fig. 5), together establishing SbbHLH168 as both necessary and sufficient for its salt-tolerance role.

### SbbHLH168 confers salt tolerance through coordinated multi-pathway mechanisms

SbbHLH168-OE plants accumulated less Na⁺ and maintained a lower Na⁺/K⁺ ratio than WT under salt stress in both Arabidopsis and sorghum (Figs. 3C-D, 4E-G), while silenced groups showed the opposite pattern (Fig. 5F-H). Concomitantly, relative electrolyte leakage decreased significantly in OE plants (Fig. 3E). The role of bHLH factors in Na⁺/K⁺ regulation is conserved across species, though the downstream transporters they target differ. In rice, OrbHLH001 from wild rice activates *OsAKT1* to regulate Na⁺/K⁺ balance (Chen et al. 2013), and OsbHLH044 maintains ion homeostasis by regulating *OsHKT1;3*, *OsHAK7*, *OsSOS1*, and *OsNHX2* (Alam et al. 2022). In pear, the PbbHLH62/PbVHA-B1 module controls vacuolar ATPase activity, coupling Na⁺/K⁺ homeostasis with ROS removal (Qiao et al. 2024). In pepper, CabHLH035 enhances salt tolerance by modulating ion homeostasis and proline accumulation (Zhang et al. 2022). Collectively, these studies—spanning monocots and dicots, wild and domesticated species—establish bHLH-mediated ion regulation as an evolutionarily deep salt-adaptation strategy. SbbHLH168 extends this conserved module into the Andropogoneae clade. Whether it directly activates specific ion transporter genes, as OrbHLH001 and OsbHLH044 do in rice, or acts through intermediary regulators, remains to be determined.

SbbHLH168-OE plants also accumulated less ROS under salt stress, as shown by reduced NBT and DAB staining intensity (Fig. 3A). This result is consistent with bHLH factors in other species that upregulate antioxidant enzymes to mitigate oxidative damage (Waseem et al. 2019). Additionally, *SbBGLU22*, a β-glucosidase gene implicated in ABA release from conjugated storage forms (Song et al. 2022a; Zheng et al. 2023), was significantly upregulated in SbbHLH168-OE under salt stress (Fig. 7A). Given the central role of ABA in orchestrating stress-responsive gene expression (Pan et al. 2020), *SbBGLU22* may link SbbHLH168 to ABA-mediated stress signaling, though whether this connection is direct or indirect remains to be determined.

The connection between SbbHLH168 and lignin biosynthesis represents the most unexpected finding of this study. The lignin pathway genes (*SbC4H*, *SbF5H* and *SbCOMT1*) were significantly upregulated in SbbHLH168-OE under salt stress, with *SbF5H* showing the strongest induction (Fig. 7A). Lignin content increased in all groups after salt treatment, but the increase was significantly greater in SbbHLH168-OE than in WT (Fig. 7B). Lignin reinforces the apoplastic barrier at Casparian bands, blocking Na⁺ diffusion into the xylem and protecting aboveground tissues from ion toxicity (Cui et al. 2021; Wu et al. 2019). SbbHLH168 thus deploys targeted lignin deposition as a structural component of its salt-tolerance mechanism.

This lignin-salt tolerance connection has precedent but also limitations in the existing literature. In *Lilium pumilum*, the BEL1-like protein LpBLH3 enhances salt tolerance by upregulating lignin biosynthesis genes (Wan et al. 2025)—consistent with our observations, though LpBLH3 belongs to a distinct transcription factor family. In rice, OsbHLH034 promotes lignin accumulation through jasmonate signaling, critically, increases salt sensitivity upon overexpression (Onohata et al. 2020). This contradicts the SbbHLH168 phenotype: both are bHLH proteins that drive lignin biosynthesis, yet they produce opposite salt-tolerance outcomes. Two non-mutually exclusive factors may explain this contradiction. First, the extremely low pairwise alignment score between SbbHLH168 and OsbHLH168 (50.00)—the lowest among all Poaceae pairs examined, comparable to non-Poaceae outgroups—indicates that these proteins have diverged substantially outside the core bHLH domain. Such divergence in regulatory and interaction surfaces likely rewires their downstream target networks. Second, the spatial distribution of lignin deposition is probably decisive: targeted lignification of Casparian bands in endodermal cells restricts apoplastic Na⁺ entry and is beneficial (as we propose for SbbHLH168), whereas ectopic lignification of cortical or epidermal cells—which may occur under OsbHLH034 overexpression—rigidifies cell walls, impairs root growth and nutrient uptake, and exacerbates stress sensitivity. The RSS3–bHLH–JAZ complex in rice, which modulates root cell wall plasticity and lignin deposition (Toda et al. 2013), independently demonstrates that bHLH-containing complexes mediate cell wall remodeling with context-dependent phenotypic outcomes.

To our knowledge, SbbHLH168 is the first bHLH transcription factor demonstrated to coordinate ion homeostasis and lignin biosynthesis as an integrated salt-tolerance mechanism. Other characterized bHLH proteins regulate ion transport (OrbHLH001, OsbHLH044, PbbHLH62, CabHLH035) or lignin biosynthesis (OsbHLH034) individually, but none has been shown to control both processes simultaneously under a single stress condition. This dual functionality distinguishes SbbHLH168 within the bHLH family. How this coordination is achieved remains an open question. SbbHLH168 may independently bind promoters of both ion transporter genes and lignin biosynthesis genes; alternatively, its structural reinforcement of Casparian bands may passively limit apoplastic Na⁺ entry, creating an indirect structural–physiological coupling rather than strictly parallel transcriptional regulation. A third model involves partner-dependent specificity: the composition of transcriptional complexes containing SbbHLH168—potentially incorporating co-regulators active in phenylpropanoid metabolism—may determine which gene class is activated. Distinguishing these models will require genome-wide binding site mapping (ChIP-seq) combined with transcriptomic profiling under salt stress.

### SbbHLH168-SbbHLH35 dimerization is likely essential for function

bHLH factors commonly regulate transcription as homo- or heterodimers (Hernandez et al. 2007), and dimerization can switch their target gene specificity and subcellular localization (Zuo et al. 2023). In Arabidopsis, bHLHIVc-bHLH121 heterodimerization promotes nuclear accumulation of bHLH121 and activation of *FIT*, to regulate iron uptake (Lei et al. 2020), and the antagonistic BEE–IBH1 pair controls stress tolerance through dimerization-dependent mechanisms (Moreno et al. 2018). Here, Y2H and BiFC assays confirmed a specific nuclear interaction between SbbHLH168 and SbbHLH35 (Fig. 6). While bHLH35 orthologs have been linked to cold and drought tolerance (Dong et al. 2014; Jiang et al. 2019), our results extend SbbHLH35's functional range into salt stress adaptation.

Whether the SbbHLH168-SbbHLH35 heterodimer alters DNA-binding specificity relative to either homodimer, or enables synergistic activation of downstream targets (for example, ion homeostasis genes versus lignin biosynthesis genes), remains to be determined. If the heterodimer recognizes distinct promoter elements in each pathway, this would provide a parsimonious mechanistic explanation for the dual-function phenotype. Testing this hypothesis will require comparative ChIP-seq of SbbHLH168 in WT and SbbHLH35-knockdown backgrounds. That SbbHLH85 interacts with PHF1 rather than a bHLH partner (Song et al. 2022b) further illustrates the diversity of interaction strategies within the sorghum bHLH network.

## Conclusion

In summary, this study identified SbbHLH168 as a key transcription factor in sorghum that significantly enhances salt tolerance by reducing Na⁺ accumulation, maintaining a low Na⁺/K⁺ ratio, and alleviating oxidative stress. Notably, SbbHLH168 upregulates key lignin biosynthesis genes (*SbC4H*, *SbF5H*, *SbCOMT1*) to increase lignin content, potentially enhancing salt tolerance by strengthening cell wall barriers and limiting ion leakage under stress. Furthermore, the interaction between SbbHLH168 and SbbHLH35 reveals that heterodimerization is crucial for its regulatory function. This discovery provides a valuable gene resource for direct utilization in crop salt tolerance molecular design and expands the functional boundaries of the bHLH family.

## Materials and methods

### Phylogenetic analysis of SbbHLH168

The genome sequence and amino acid sequence of SbbHLH168 (SORBI_3003G102200) was obtained from *Ensembl Plants*. The neighbor-joining (NJ) phylogenetic tree was constructed using MEGA6 software with 1000 bootstrap repetitions. The bHLH168 homologous protein sequences were retrieved from 15 gramineous species (*Sorghum bicolor*, *Panicum virgatum*, *Panicum hallii*, D*igitaria exilis*, *Setaria italica*, *Setaria viridis*, *Paspalum vaginatum*, *Zea mays*, *Miscanthus lutarioriparius*, *Eragrostis curvula*, *Zizania palustris*, *Oryza sativa*, *Lolium multiflorum*, *Triticum aestivum*, *Hordeum vulgare*) and 3 non-gramineous species (*Phoenix dactylifera*, *Colocasia esculenta*, *Asparagus officinalis*).

### Plant materials and growth conditions

*Arabidopsis thaliana* Col-0 and *Sorghum bicolor* BTx623 were used as the wild-type backgrounds for this study. The *SbbHLH168*-overexpressing Arabidopsis lines were generated via floral dip transformation of Col-0 using the binary vectors pCAMBIA1300-35S:SbbHLH168-GFP and pCAMBIA3301-proSbbHLH168:GUS. For sorghum transformation, *SbbHLH168*-overexpressing hairy roots in the BTx623 background were obtained through *Rhizobium rhizogenes* (strain K599)-mediated transformation, with the construct pCAMBIA2300-35S:SbbHLH168-GFP. Virus-induced gene silencing (VIGS) was employed to generate *SbbHLH168* knockdown groups using the pTRV2-SbbHLH168 vector, described by Zhang et al. (2017). Plants exhibiting > 50% reduction in transcript level (qRT-PCR) were retained for phenotyping. All plants were cultivated under identical conditions (16 h light/8 h dark photoperiod, 22–28 °C, 60–70% relative humidity).

### Salt stress treatment

For short-term salt treatments, surface-sterilized (2% NaClO) sorghum seeds were hydroponically grown until the three-leaf-one-heart stage, then transferred to ^1^/_2_ Hoagland solution containing 150 mM NaCl. Root and leaf tissues were harvested at 0, 3, 6, 12, 24, 48 and 72 h, snap-frozen in liquid nitrogen, and stored at − 80 °C.

For long-term salt treatments in Arabidopsis, two independent experimental systems were used. For root length assays, Col-0 and transgenic seeds were grown on ½ MS medium containing 0, 50, 100 or 150 mM NaCl for 7 days. For phenotypic and physiological analyses, 2-week-old soil-grown seedlings were irrigated with 0 or 100 mM NaCl for an additional 7 days. For sorghum, BTx623 and transgenic groups were treated with 0 or 150 mM NaCl for 7 days. At the end of the treatment period, roots and leaves were sampled, weighed, and immediately processed for physiological and molecular assays.

### Physiological parameter assays

Fresh samples were rinsed with ddH₂O, blotted dry and weighed for fresh weight (FW). For Arabidopsis, plants of similar size and vigor from each line were grouped (5 plants as one biological replicate), oven-dried to constant weight, and weighed for dry weight (DW). Na⁺ and K⁺ contents were determined with a flame photometer. Fresh tissue (0.3 g) was homogenized in 10 mL ddH₂O for 2–3 h, filtered, brought to 25 mL. Na⁺ and K⁺ concentrations were quantified using flame photometry, and the Na⁺/K⁺ ratio was calculated. Relative electrical conductivity (REC) was measured as (initial conductivity / total conductivity after boiling) × 100% using 0.2 g leaves immersed in 6 mL ddH₂O. Lignin was quantified in lyophilised sorghum tissue. Samples were digested in acetyl-bromide:acetic acid (1:3, v/v) and absorbance read at 280 nm against a Sigma alkali lignin standard curve. All assays were performed with ≥ 3 biological replicates per treatment group per experiment, and each entire experiment was independently repeated three times to ensure reproducibility and to minimize random or experimental errors. The data presented are representative of three independent experiments.

### Histochemical staining

H₂O₂ and O₂⁻ were visualised with 3,3'-diaminobenzidine (DAB, pH 5.8) and nitro-blue tetrazolium (NBT, 0.5 mg mL^⁻1^ in 50 mM PBS pH 7.4), respectively. Leaf segments were vacuum-infiltrated, incubated 8 h (DAB) or 3 h (NBT) at 28 °C in darkness, cleared in boiling 80% ethanol and photographed.

For GUS assays, a 2.0-kb SbbHLH168 promoter was cloned into pCAMBIA3301-UBI-GUS vector and transformed into Arabidopsis. Tissues from various developmental stages (roots, leaves, flowers, siliques) were stained with X-Gluc solution (1 mM X-Gluc in 0.1 M PBS, pH 7.0) overnight at 37 °C. Chlorophyll was subsequently removed by destaining in 80% ethanol, and stained tissues were photographed.

### Quantitative Real-Time PCR (qRT-PCR)

Total RNA was extracted from the sorghum leaves using Trizol reagent (Huayueyang, Beijing, China) following the manufacturer's protocol. cDNA was synthesized using the Evo M-MLV Reverse Transcription Kit (Accurate Biology, Hunan, China). qPCR was performed using SYBR Green Master Mix (Accurate Biology, Hunan, China) on a real-time PCR system. The SbActin (sorghum) or Atactin (Arabidopsis) served as the internal reference. Relative gene expression levels were calculated by the 2^(–ΔΔCt) method.

### Subcellular localization

The pCAMBIA1300-35S:SbbHLH168-GFP expression vector was introduced into Agrobacterium GV3101. Bacterial suspensions were infiltrated into leaves of *N. benthamiana*. GFP fluorescence signals were visualized 48 h post-infiltration using a Zeiss LSM 880 confocal laser scanning microscope.

### Protein–protein interaction

Yeast two-hybrid (Y2H) assays were performed using pGBKT7-SbbHLH168 as bait and pGADT7-SbbHLH35 as prey. Co-transformed Y2HGold cells were plated on SD/-Leu/-Trp (DDO) and SD/-Leu/-Trp/-His/-Ade (QDO) media containing X-α-Gal and incubated at 30 °C for 3 d.

For BiFC, SbbHLH168 and SbbHLH35 were fused to the C-terminal (cYFP) and N-terminal (nYFP) fragments of YFP, respectively. Constructs were co-expressed in *N. benthamiana* leaves. Reconstituted YFP fluorescence signals were observed using confocal microscopy 48 h post-infiltration.

### Statistical analysis

Data are means ± standard deviation (SD) of at least three independent experiments. Data analysis was performed using GraphPad Prism version 10 (GraphPad Software, Boston, MA, USA). Differences among samples within treatment groups were assessed using the ANOVA (one-way or two-way) with Tukey's test, and differences were considered significant at *P* < 0.05. Statistically significant differences are indicated by different letters or asterisks (ns indicates the *p*-value > 0.05, * indicates the *p*-value < 0.05, ** indicates the *p*-value < 0.01, *** indicates the *p*-value < 0.0005, **** indicates the *p*-value < 0.0001).

## Supplementary Information

Supplementary Material 1.

Supplementary Material 2.

## Data Availability

All data generated or analyzed during this study are included in this article and/or its supplementary information files.

## Abbreviations

TFs: Transcription factors
bHLH: basic helix-loop-helix
VIGS: Virus-induced gene silencing
ROS: Reactive oxygen species
FW: Fresh weight
DW: Dry weight
REC: Relative electrical conductivity
DAB: 3,3'-Diaminobenzidine
NBT: Nitro blue tetrazolium
GFP: Green fluorescent protein
qRT-PCR: Quantitative real-time polymerase chain reactions
*N. benthamiana*: *Nicotiana benthamiana*
