# Supplementary material for: The transcription factor SbbHLH168 enhances salt tolerance by coordinating ion homeostasis and lignin content in sorghum
Source: Stress Biol. 2026 Jul 21;6(1):51. doi: 10.1007/s44154-026-00330-4 (PMC13388629; doi:10.1007/s44154-026-00330-4)
Supplement: Supplementary file 1 — Supplementary Material 1. [file 44154_2026_330_MOESM1_ESM.pdf]

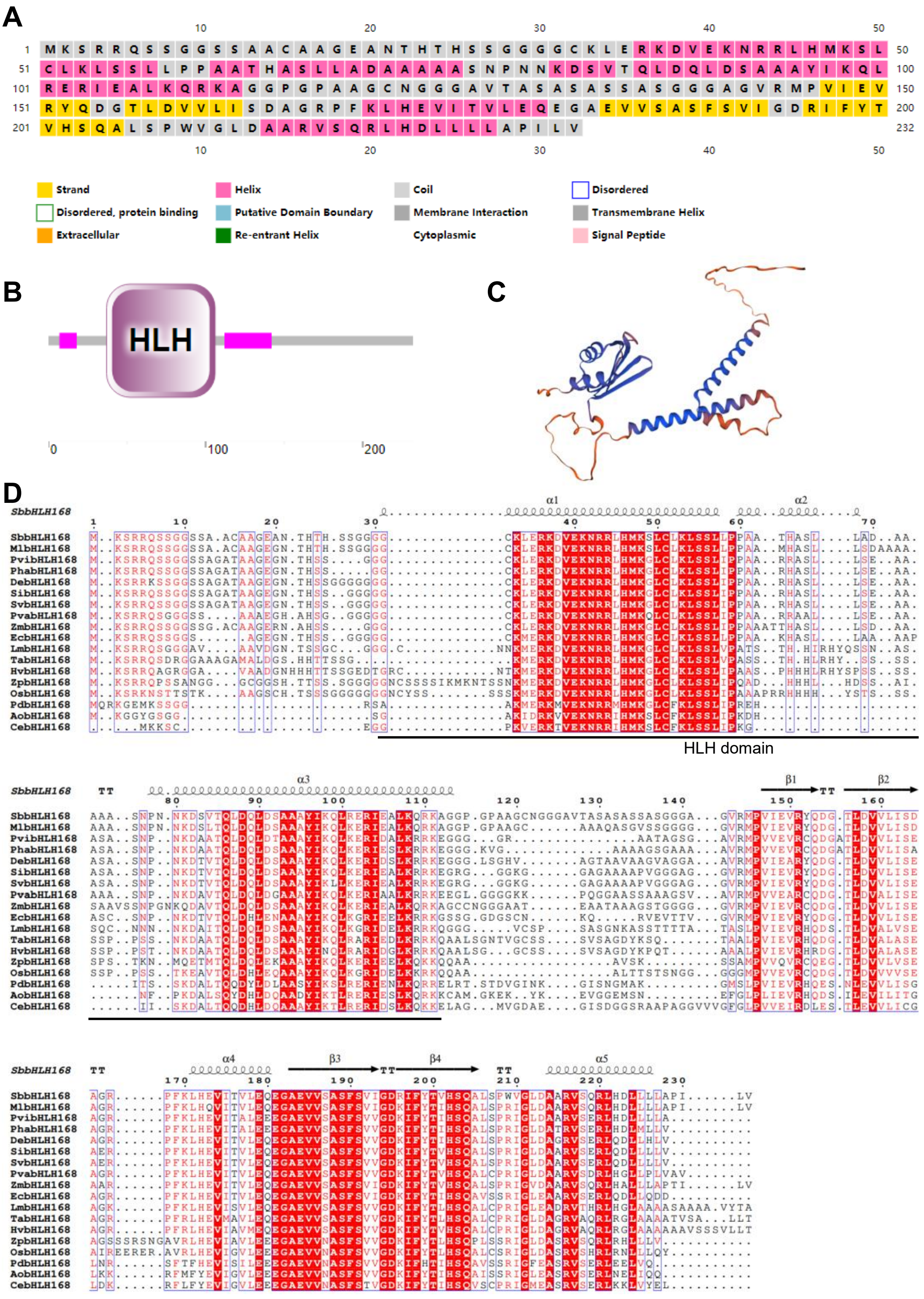


**Fig. S1. Sequence conservation and structural architecture of SbbHLH168. (A)** Secondary structure analysis of SbbHLH168. **(B)** Structural domain analysis of SbbHLH168. **(C)** Tertiary structure analysis of SbbHLH168. **(D)** Multiple sequence alignment of SbbHLH168 and its homologs from the 18 plant species represented in Figure 1A, generated using ESPript 3.0 (https://espript.ibcp.fr/ESPript/ESPript/).

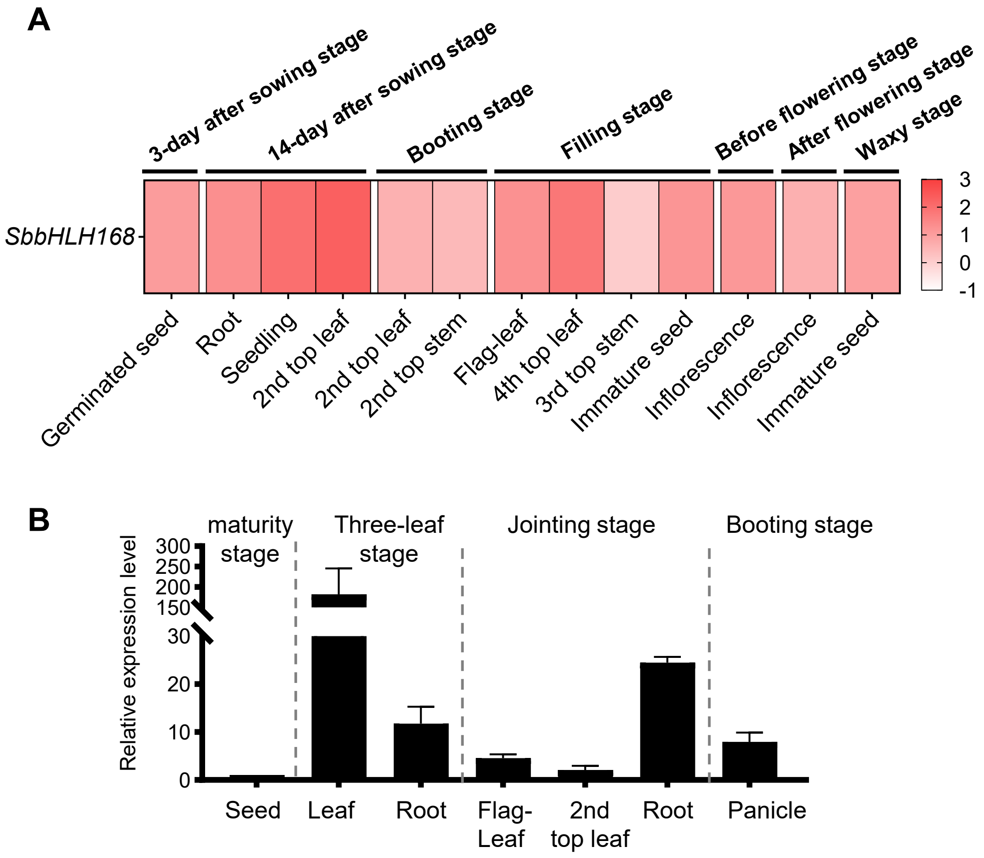


**Fig. S2. Spatiotemporal expression pattern of *SbbHLH168* in sorghum. (A)** Heatmap of *SbbHLH168* expression [$\log_{10}^{(TPM+1)}$] across tissues and developmental stages based on publicly available sorghum transcriptome data (Chen et al. 2025). **(B)** Relative expression levels of *SbbHLH168* in seed (maturity stage), leaf and root (three-leaf stage), flag leaf, 2nd top leaf and root (jointing stage), and panicle (booting stage), determined by qRT-PCR. Error bars indicate mean ± SD, n = 3.

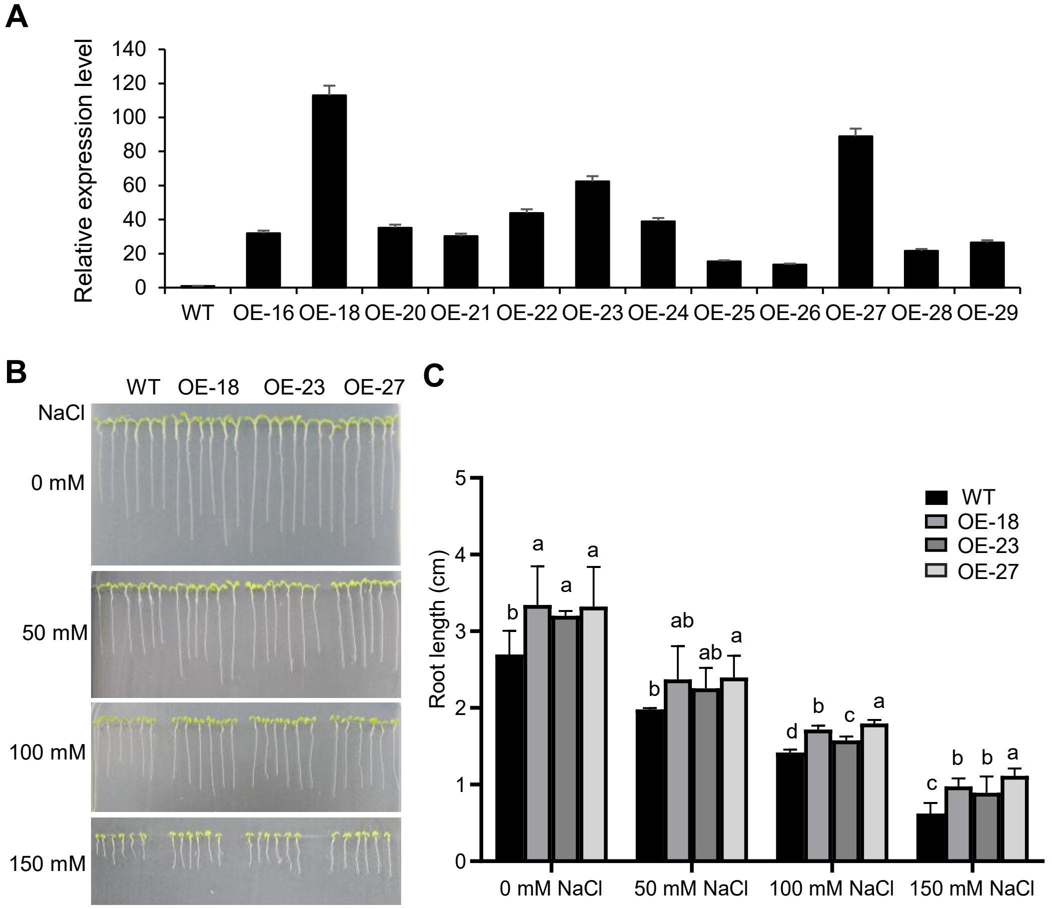


**Fig. S3. Detection of *SbbHLH168* expression levels and root length sensitivity in SbbHLH168-OE transgenic Arabidopsis.** **(A)** Identification of SbbHLH168 expression levels in SbbHLH168-OE transgenic Arabidopsis plants. **(B)** Root phenotypes of WT and SbbHLH168-OE lines that were grown on vertical $^{1}/_{2}$MS plates supplied with the indicated concentration of NaCl for 7 days. **(C)** Quantitation of the root length of the salt-treated plants shown in (B). Error bars indicate mean± standard deviation (SD), n=6. Statistically significant differences are indicated by different letters (Student's t-test, $p < 0.05$).

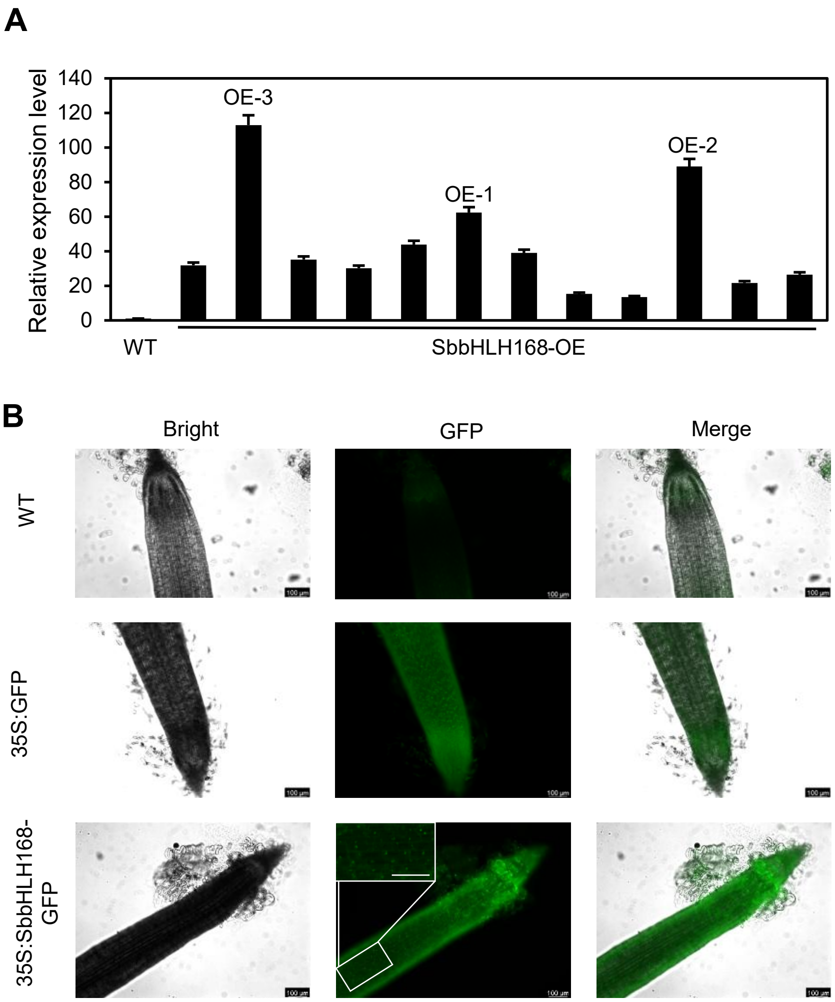


**Fig. S4. Identification of SbbHLH168-OE in sorghum. (A)** Relative expression levels of *SbbHLH168* in WT and OE plants was determined by qRT-PCR. Based on the fold increase in bHLH168 expression, these transgenic plants were artificially divided into three gradient groups: Group 1 (SbbHLH168-OE-1), with relative expression levels ranging from 60 to 80; Group 2 (SbbHLH168-OE-2), with relative expression levels ranging from 90 to 110; and Group 3 (SbbHLH168-OE-3), with relative expression levels ranging from 110 to 130. **(B)** Fluorescence validation of SbbHLH168-GFP in sorghum roots. GFP fluorescence was observed in roots expressing the 35S:GFP empty vector and 35S:SbbHLH168-GFP fusion protein. The white box indicates the root elongation zone.

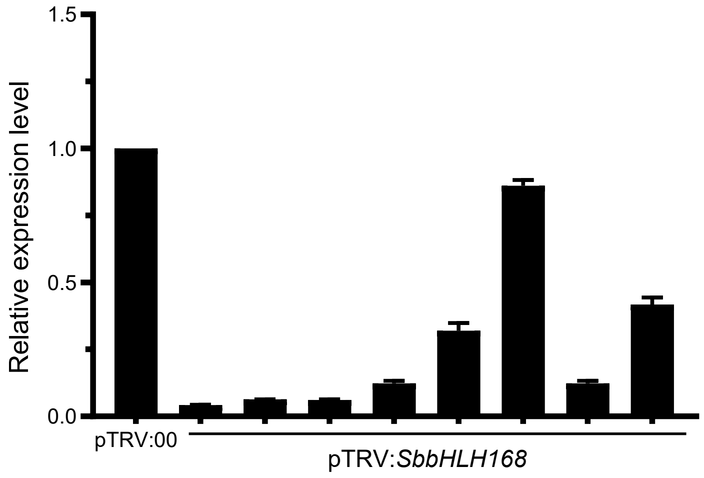


**Fig. S5. Expression level of *SbbHLH168* in some pTRV:*SbbHLH168* silenced plants**. pTRV:00 (empty vector)-infiltrated sorghum served as the control. Based on the strength of the silencing efficiency, these transient plants were artificially divided into three gradient groups, namely pTRV:*SbbHLH168*-1 (strong silencing group): relative expression < 0.05; pTRV:*SbbHLH168*-2 (moderate silencing group): relative expression 0.05–0.10; pTRV:*SbbHLH168*-3 (mild silencing group): relative expression 0.10–0.15.

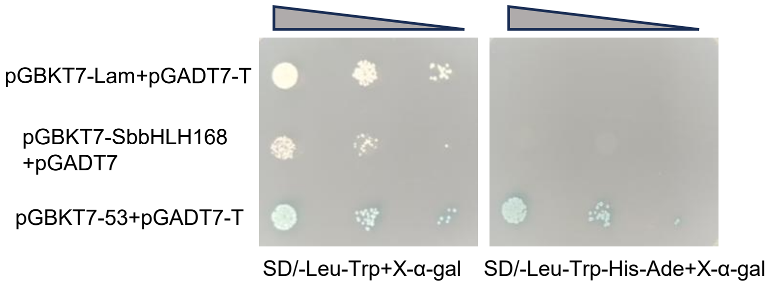


**Fig. S6. Detection of the self-activation activity of SbbHLH168 using the yeast two-hybrid assay.** The yeast cells were co-transformed with pGBKT7-SbbHLH168 and pGADT7 plasmids, then inoculated onto SD/-Leu/-Trp/X-α-gal and SD/-Leu/-Trp/-His/-Ade/X-α-gal medium for 3 days. pGBKT7-53 + pGADT7-T served as the positive control group, while pGBKT7-Lam + pGADT7-T served as the negative control group.

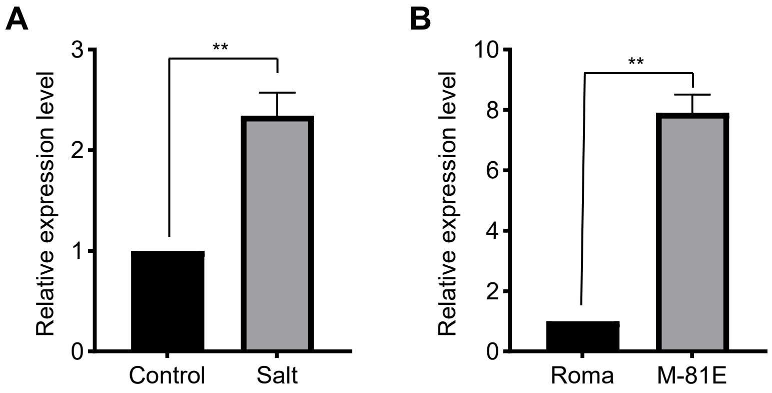


**Fig. S7. *SbbHLH35* expression responds to salt stress. (A)** Relative expression levels of *SbbHLH35* in WT roots after 48 h of 150 mM NaCl treatment, determined by qRT-PCR. **(B)** Comparison of *SbbHLH35* basal expression between the salt-sensitive cultivar Roma and the salt-tolerant cultivar M-81E (Yang et al. 2018a). Error bars indicate mean ± SD. $^{**}P < 0.01$ (Student's t-test), $n = 3$.
